# RETRACTION: Early Versus Deferred Endovenous Sclerotherapy of Superficial Venous Reflux in Patients With Venous Ulceration

**DOI:** 10.1111/iwj.70603

**Published:** 2025-04-21

## Abstract

**RETRACTION:** LiuJ. and GuoQ. , “Early Versus Deferred Endovenous Sclerotherapy of Superficial Venous Reflux in Patients With Venous Ulceration,” International Wound Journal 21, no. 3 (2024): e14445, 10.1111/iwj.14445.37845810 PMC10895194

The above article, published online on 16 October 2023, in Wiley Online Library (http://onlinelibrary.wiley.com/), has been retracted by agreement between the journal Editor in Chief, Professor Keith Harding; and John Wiley & Sons Ltd. Following an investigation by the publisher, all parties have concluded that this article was accepted solely on the basis of a compromised peer review process. In addition, the investigation found that the authors provided incomplete informed consent information for the study. The editors have therefore decided to retract the article. The authors did not respond to our notice regarding the retraction.

**RETRACTION:** J. Liu and Q. Guo , “Early Versus Deferred Endovenous Sclerotherapy of Superficial Venous Reflux in Patients With Venous Ulceration,” International Wound Journal 21, no. 3 (2024): e14445, 10.1111/iwj.14445.37845810 PMC10895194

The above article, published online on 16 October 2023, in Wiley Online Library (http://onlinelibrary.wiley.com/), has been retracted by agreement between the journal Editor in Chief, Professor Keith Harding; and John Wiley & Sons Ltd. Following an investigation by the publisher, all parties have concluded that this article was accepted solely on the basis of a compromised peer review process. In addition, the investigation found that the authors provided incomplete informed consent information for the study. The editors have therefore decided to retract the article. The authors did not respond to our notice regarding the retraction.

